# Supplementary material for: The copper chaperone ATOX1 exhibits differential protein-protein interactions and contributes to skeletal myoblast differentiation
Source: bioRxiv. 2025 Jul 10:2025.07.08.663731. Preprint. [Version 1] doi: 10.1101/2025.07.08.663731 (PMC12265579; doi:10.1101/2025.07.08.663731)
Supplement: Supplement 2 [file NIHPP2025.07.08.663731v1-supplement-2.pdf]

bioRxiv preprint doi: https://doi.org/10.1101/2025.07.08.663731; this version posted July 10, 2025. The copyright holder for this preprint (which was not certified by peer review) is the author/funder, who has granted bioRxiv a license to display the preprint in perpetuity. It is made available under aCC-BY 4.0 International license.

Supplemental Table 1

Company	Antibody	Catalog #	Dilution
Wang et al PLOS One, 2012	ATP7A	N/A	1:1000
Proteintech	ATOX1	Catalog#: 22641-1-AP	1:1000
Lab made	eMyHC	N/A	1:10
Lab made	Flag	N/A	1:1000
Cell Signaling Technology	Histone H3	4499s	1:4000
Cell Signaling Technology	GAPDH	5174S	1:1000
Santa Cruz	CCS	sc-55561	1:1000
Cell Signaling Technology	MEK1/2	9122	1:1000
Lab made	ATP7A	N/A	1:1000
Cell Signaling Technology	HSP90	4877S	1:4000
Proteintech	PLOD2	21214-1-AP	1:1000
Proteintech	Scamp3	67932-1-Ig	1:1000
Proteintech	CRIP2	14801-1-AP	1:1000
Proteintech	SYNCRIP	14024-1-AP	1:1000
Jackson Laboratory	Mouse-HRP	115-035-003	1:10000
Jackson Laboratory	Rabbit-HRP	111-035-003	1:10000
Cell Signaling Technology	Streptavidin-HRP	3999S	
Jackson Laboratory	Mouse-Alexa Fluor 488	715-545-151	1:500

bioRxiv preprint doi: https://doi.org/10.1101/2025.07.08.663731; this version posted July 10, 2025. The copyright holder for this preprint (which was not certified by peer review) is the author/funder, who has granted bioRxiv a license to display the preprint in perpetuity. It is made available under aCC-BY 4.0 International license.

Supplemental Figure 1

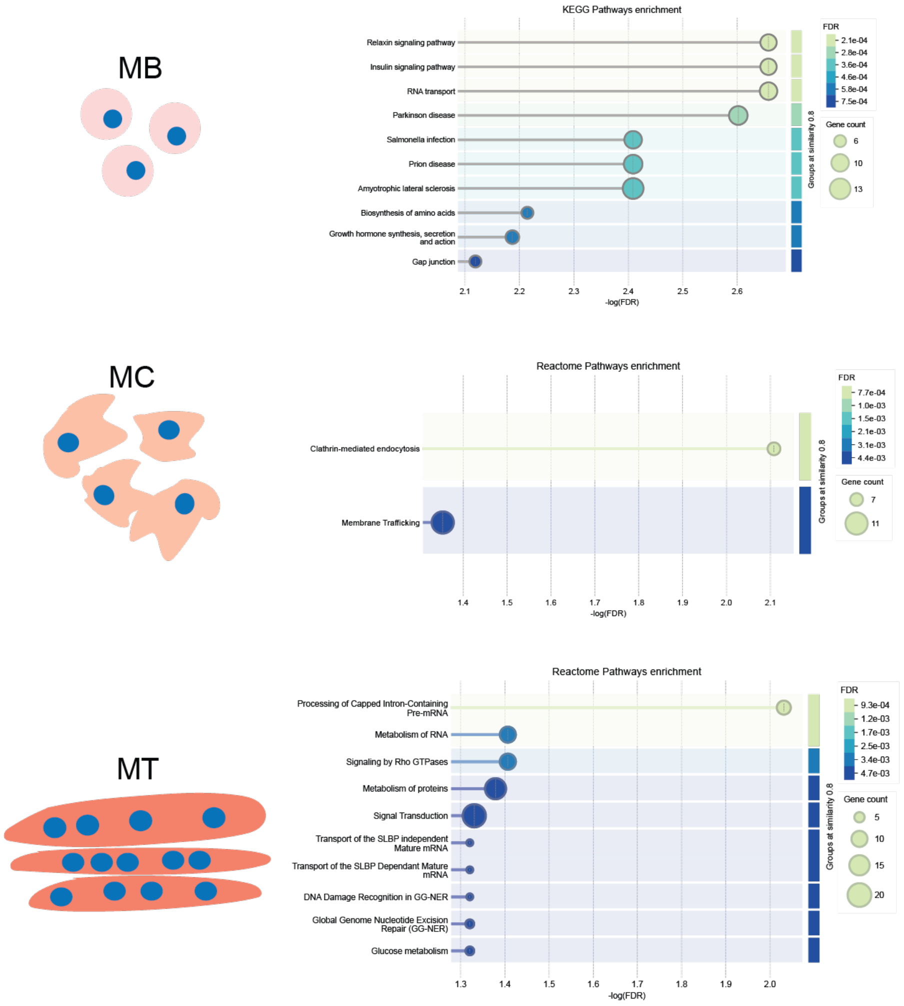

Supplemental Figure 1: Additional functional annotation of ATOX1 proximal proteins
Shown are enriched KEGG pathway in myoblasts (MB) and Reactome pathways in myocytes (MC) and myotubes (MT). No KEGG pathway enrichment was detected in MC or MT and no Reactome pathway enrichment was detected in MB. Pathway analysis was performed using the STRING database.

bioRxiv preprint doi: https://doi.org/10.1101/2025.07.08.663731; this version posted July 10, 2025. The copyright holder for this preprint (which was not certified by peer review) is the author/funder, who has granted bioRxiv a license to display the preprint in perpetuity. It is made available under aCC-BY 4.0 International license.

Supplemental Figure 2

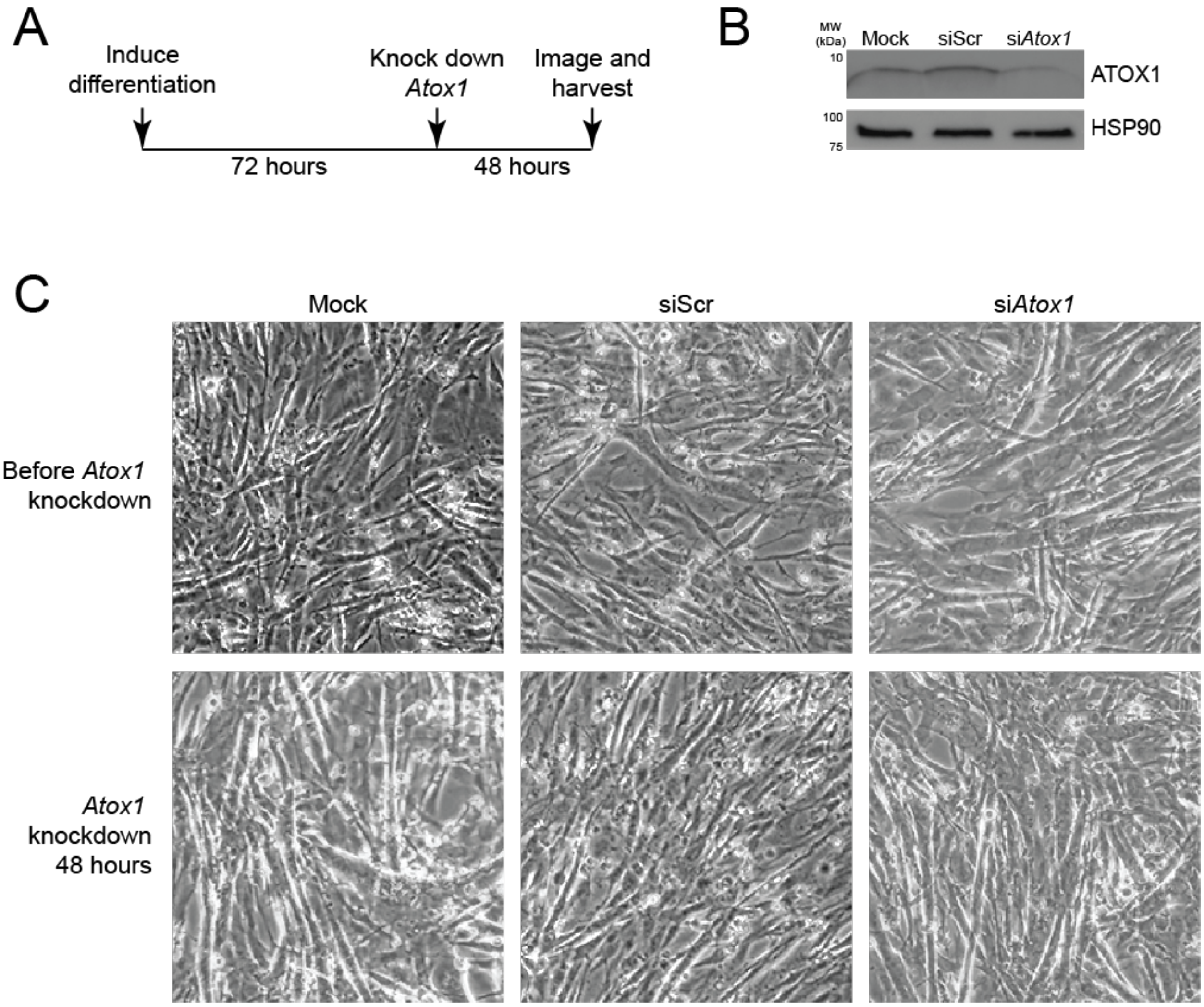

Supplemental Figure 2: No overt phenotype cause by ATOX1 deficiency in fully differentiated myotubes. ***A)*** Schematic of experiment. Briefly, myoblasts were allowed to differentiate for 72 hours, transfected with control or *Atox1* targeting siRNA (siAtox1), and harvested 48 hours later. ***B)*** Immunoblot probed with an antibody to ATOX1 showing reduced ATOX1 protein in *Atox1* knockdown myotubes compared to mock transfected (Mock) or non-targeting siRNA (siScr) control myotubes. Antibody targeting HSP90 was used as a loading control. ***C)*** Phase contrast images of early (top) and mature (bottom) myotubes in cells prior to transfection (top) and 48 hours after transfection (bottom) showing no overt phenotype in myotubes transfected with siAtox1.
